# Surface modifications of biomaterials in different applied fields

**DOI:** 10.1039/d3ra02248j

**Published:** 2023-07-10

**Authors:** Xi Hu, Teng Wang, Faqi Li, Xiang Mao

**Affiliations:** a State Key Laboratory of Ultrasound in Medicine and Engineering College of Biomedical Engineering, Chongqing Medical University Chongqing 400016 P. R. China maox@cqmu.edu.cn; b Chongqing Key Laboratory of Biomedical Engineering, College of Biomedical Engineering, Chongqing Medical University Chongqing 400016 P. R. China

## Abstract

Biomaterial implantation into the human body plays a key role in the medical field and biological applications. Increasing the life expectancy of biomaterial implants, reducing the rejection reaction inside the human body and reducing the risk of infection are the problems in this field that need to be solved urgently. The surface modification of biomaterials can change the original physical, chemical and biological properties and improve the function of materials. This review focuses on the application of surface modification techniques in various fields of biomaterials reported in the past few years. The surface modification techniques include film and coating synthesis, covalent grafting, self-assembled monolayers (SAMs), plasma surface modification and other strategies. First, a brief introduction to these surface modification techniques for biomaterials is given. Subsequently, the review focuses on how these techniques change the properties of biomaterials, and evaluates the effects of modification on the cytocompatibility, antibacterial, antifouling and surface hydrophobic properties of biomaterials. In addition, the implications for the design of biomaterials with different functions are discussed. Finally, based on this review, it is expected that the biomaterials have development prospects in the medical field.

## Abstract 2

Biomaterial implantation into the human body plays a key role in the medical and biological field. It increases the life expectancy, reduces the rejection reaction and risk in infections.

## Graphical abstract

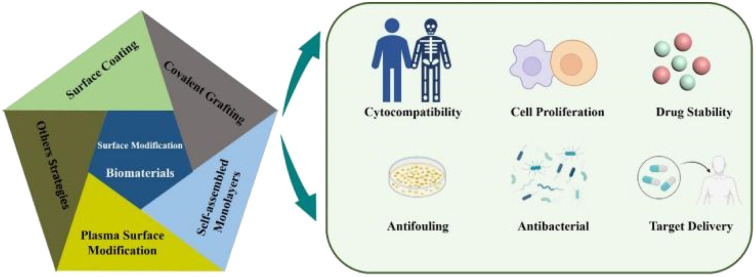

## Introduction

The development of modern medicine has made it possible to regenerate and rebuild damaged human tissues and organs. Biomaterials show strong vitality and broad development prospects in different medical fields. In this case, biological materials can be divided into metal materials, ceramic materials and polymer materials.^1^ To date, a variety of materials based on polymers, ceramics and metals have been widely used in tissue engineering, including micro-implants for cardiovascular purposes and macroscopic devices in bone tissues, among many other applications.^2^ Because of their excellent mechanical properties (high strength toughness and fatigue resistance) and chemical properties, metal-based biomaterials have been successfully applied as artificial implants in the biomedical engineering field.^3–5^ The clinical application of ceramic and polymer materials as implants has also been widely studied. Even so, the utilized biological materials cannot avoid the occurrence of biocompatibility, protein adsorption, and bacterial adhesion. In this way, biocompatibility is the primary problem to be solved in surgical implantation.^6^ Traditionally, biological materials implanted in the body will inevitably be rejected by the body, and the immune mechanism will show defense against them, leading to the failure of implantation of materials.^7,8^ The growth of microorganisms in the implant leads to an increased rate of surgical infection. Most bacteria contaminating implants come from the skin surface and mucous membrane. These bacteria adhere to the surface of implant materials and proliferate to form biofilms, leading to infection finally.^9^ In this case, biofilms can damage the tissue surrounding the implant, resulting in poor vascularization, loosening, detachment and even dislocation of the implant material.^10^ Furthermore, the biofilm formation process occurs *via* two stages: one is reversible interaction between bacteria and the surface of the biomaterial and the other one is an irreversible process, *i.e.* proteins on the surface of bacteria and the surface of the biomaterial could bind together to produce specific and non-specific interactions.^11^ Biological contamination triggers foreign body reactions including nonspecific adsorption of proteins and adhesion of inflammatory cells. Protein adsorption on the surface of biomaterials plays a key role in the subsequent processes of cell behaviour and extracellular matrix (ECM) formation.^12^ The infection and inflammation are the main causes of complications and failure of biomaterial implantation, both of which are caused by the interaction between cells and biomaterials.^9^ Therefore, it is of vital importance to design medical biomaterials, which are both anti-fouling and anti-infective with highly biocompatible features. This not only significantly improves the clinical outcomes, but also reduces the financial burden of implant failure in patients.

The interaction of biomaterials with tissues is determined by their surface properties. Therefore, surface modification is considered as an important means to improve the biological properties. The surface modification of implanted biomaterials is an effective way to improve biocompatibility and reduce the incidence of associated infections.^13^ It is common to modify the surface of biomaterials without changing the properties of the substrates on a micro or nano scale.^14,15^ Surface modification provides controllable and programmable surface properties for biomaterials at the same time. It provides effective physical or chemical properties to make implanted biomaterials “more compatible” inside the human body.^16,17^ Because of their advantages and disadvantages, these techniques can be used individually or in combination.^18^ In this review article, the effects of surface modification on the properties of biomaterials were discussed from the perspective of chemical treatment and its combination with physics.

**Fig. 1:**
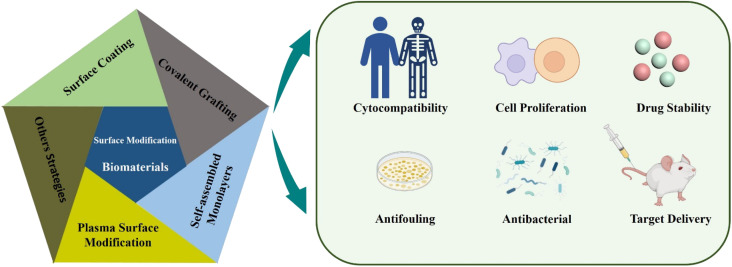
Schematic illustration of different techniques of surface modification of micro- and nano-scale biomaterials and the impact thereof.

There are several surface modification strategies widely used in the modification of biomaterials, such as surface coatings and synthetic films, covalent grafting, self-assembled monolayers (SAMs), and plasma treatment (Fig. 1). Recently, several researchers have found that titanium (Ti), zirconia (ZrO_2_), and polyetheretherketone (PEEK) have been used as orthopedic implants due to their excellent biocompatibility. The surface was modified with NaOH, which significantly improved the water contact angle, protein adhesion and bioactivity of these materials.^19^ Sharma *et al.* developed a stable, multifunctional, new-generation implantable urological biomaterial grafted with polyethyleneimine and poly(2-ethyl-2-oxazoline), which showed excellent antifouling performance and biocompatibility.^20^ As a coating agent, SAMs have antifouling properties on the surface of biomaterials and resist the adsorption of non-specific proteins.^21^ Among the surface modifications, hydrophobic surface is easy to cause protein adsorption, leading to biofilm formation. Researchers introduce hydrophilic materials to the surface, such as synthetic coatings or plasma treatments that produce hydrophilic functional groups that resist non-specific protein adsorption and bacterial adhesion.^22^ This review article mainly introduces several common surface modification strategies, focuses on the steps required by surface modification strategies and discusses the rationality of surface modification for biomaterials in medical applications. It includes biocompatibility, anti-infection and surface functionalization of biomaterials. In general, this kind of modification of biomaterials' surface (micro–nano scale) is beneficial for us to improve the possibility of implant surgery and reduce medical costs.

## Coating technology in surface modification processes

**Fig. 2:**
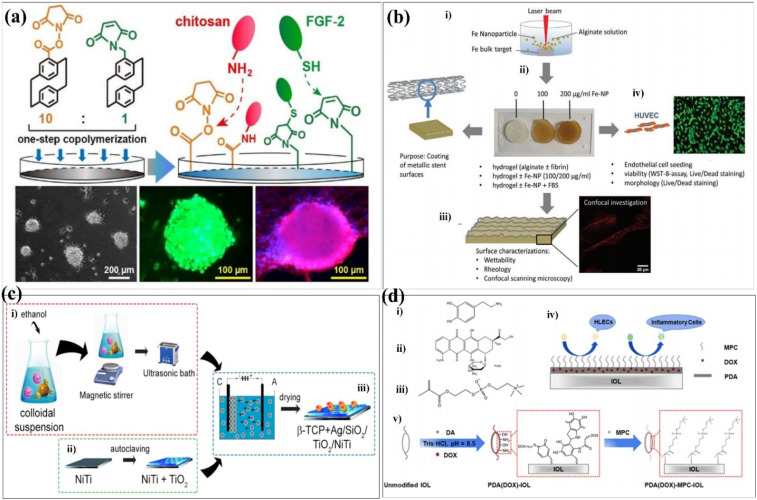
Coatings were prepared on some substrate materials to form a multifunctional surface. (a) Schematic illustration of the modification layer prepared *via* CVD copolymerization to produce 10 : 1 NHS ester to maleimide in the side groups, and the layer was modified on a stem cell culture surface. (b) Overview of the study performed. (i) Iron nanoparticles manufactured by laser ablation in an aqueous alginate solution were used to enrich (ii) alginate- and alginate-fibrin-hydrogels. (iii) Hydrogels were characterized for wettability and rheological properties by confocal microscopy and (iv) seeded with endothelial cells with (v) the purpose to coat metallic stent surfaces for better biocompatibility and antithrombotic properties. Fe = iron, NP = nanoparticle, FBS = fetal bovine serum, HUVEC = human umbilical cord endothelial cells. (c) Schematic of the manufacturing procedure to develop the multifunctional composite coatings composed of β-TCP + Ag/SiO_2_ on the TiO_2_/NiTi alloy. (i) Preparation of the colloidal suspension. (ii) Passivation of the NiTi. (iii) Coating formation. (d) (i–iii) Chemical structure formulas of DA, DOX, and MPC, respectively. (iv) Mechanism of the antiadhesive and antiproliferative PDA(DOX)-MPC coating-modified IOL. (v) Schematic illustration of the construction of the PDA(DOX)-MPC coating on the IOL surface *via* DA self-polymerization. These are reprinted with permission from Chen *et al.* (2018, *ACS Appl. Mater. Interfaces*), Richter *et al.* (2021, *Adv. Mater. Interfaces*), Dulski *et al.* (2019, *ACS Appl. Bio Mater.*), Liu *et al.* (2021, *ACS Biomater. Sci. Eng.*), respectively.

**Fig. 3:**
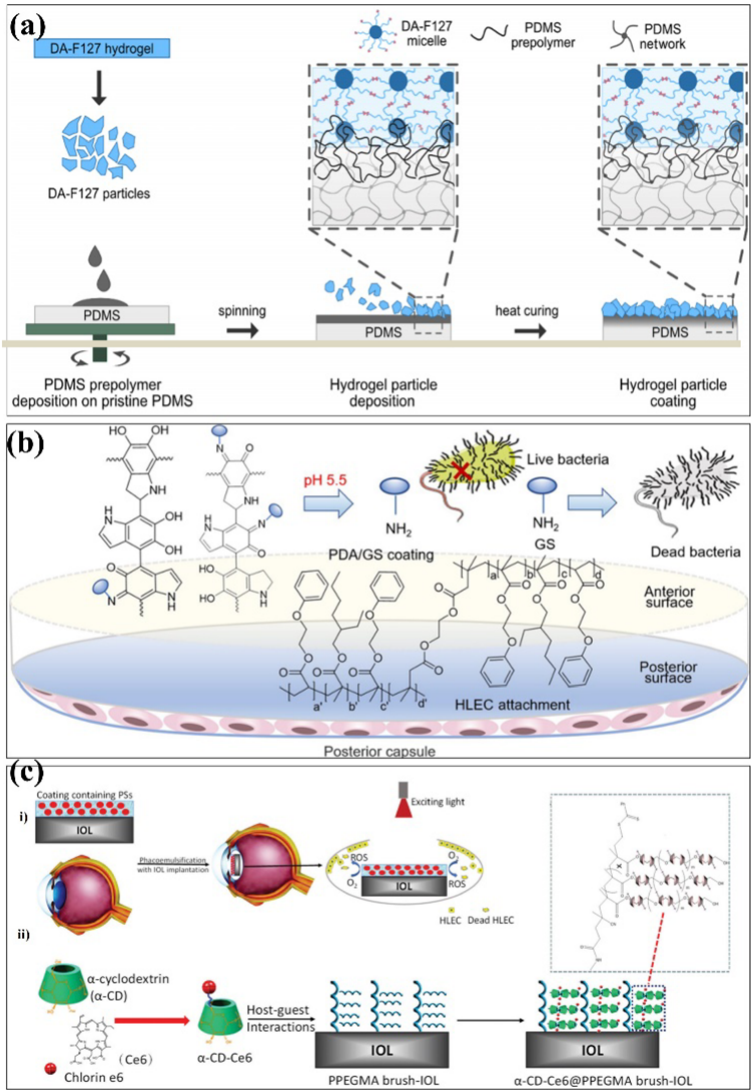
Coatings were prepared on some substrate materials to form a multifunctional surface. (a) Schematic of the proposed hydrogel particle coating onto the PDMS *via* formation of an interpenetrating polymer network. AMP modification step not included. (b) Schematic synthesis of poly(EGPEMA-*co*-EGPEA-*co*-EHMA) (PPPE) as Intraocular Lens (IOL) materials *via* free radical polymerization with dual-side heterogeneous surface modification. (c) (i) Schematic illustration of an IOL with PS-containing coating and the process of PDT. (ii) Schematic of the surface coating structure of the IOL. These are reprinted with permission from Stepulane *et al.* (2022, *ACS Appl. Bio Mater.*), Xiang *et al.* (2021, *Biomacromolecules*), Tang *et al.* (2021, *J. Mater. Chem. B*), respectively.

Surface modification techniques, in particular for biotechnological applications, were heavily pursued for creating extra physical or chemical properties as well as for designing and building potential interfaces.^23,24^ A summary of the common surface modification strategies is given in Table 1. Since the creation of 45S5 bioglass by hench, it was utilized in clinical applications as a biocompatible coating material for hard tissue replacement and regeneration.^25^ There have been several attempts to modify the makeup of bioactive glasses (BGs) in order to enhance their biological performance. Copper (Cu) exhibits antibacterial properties, and it has been reported that 45S5 bioactive glass can be fabricated by adding 5 wt% of CuO to the integrations.^26^ The investigation of the influence of Cu-containing bioactive glass on cellular behaviour has revealed that the presence of Cu induced an early differentiation of human mesenchymal stem cells (hMSCs) *via* the osteoblast phenotype, which promotes the expression of anti-inflammatory interleukin and reduces proinflammatory interleukin expressions. With the aim to produce coatings with antibacterial properties, the Cu-containing bioactive glass was used as the target material for the pulsed laser deposition (PLD) of bioactive thin films. Chen *et al.* determined a surface modification technique with excellent cohesive strength and durable coating stability, and in parallel, the modified layer exhibited a precise definition of chemical/biochemical conducts. They demonstrated a robust modification layer that was synthesized based on chemical vapor deposition (CVD) copolymerization.^27^ The copolymer modification layer's characteristics such as its adhesive strength and thermal stability demonstrated remarkable endurance (Fig. 2a). In 2018, in order to further improve the osteogenic performance of tantalum coatings, Ding *et al.* proposed a new method for developing micro/nano tantalum (MNT) with layered structures by combining plasma spraying and anodic oxidation technologies.^28^ During this process, it demonstrates that it can effectively enhance the proliferation and differentiation of human bone mesenchymal stem cells (hBMSCs) *in vitro*. Alena Richter *et al.* had proved that the Fe NP and fibrin can modify alginic acid salt and affect its wettability, surface roughness and elastic modulus. This, in turn, promotes the absorption of serum proteins, thereby promoting endothelial convergence by enhancing cell adhesion, proliferation, and vitality, thereby demonstrating a promising scaffold coating biomaterial.^29^ These synergetic effects can pave the way toward a novel strategy for the modification of various hydrogel-based biomaterials and biomaterial coatings (Fig. 2b). Dulski *et al.* prepared a colloidal suspension, composed of β-TCP and the Ag/SiO_2_ nanocomposites, which due to the electrophoretic deposition (EPD) led to the formation of structurally atypical calcium phosphosilicate coating.^30^ The purpose was to improve the functionality of NiTi alloys and extend their medical stability. The focus of this idea was to use biocompatible multifunctional coatings without affecting the functionality of the substrate (Fig. 2c). Taking advantage of self-polymerization of DA, a multifunctional coating of polydopamine(doxorubicin)-hydrophilic 2-methacryloyloxyethyl phosphorylcholine (PDA (DOX)-MPC) was constructed and modified on the intraocular lens (IOL) surface successfully. This coating was investigated by a series of experiments *in vitro* and *in vivo*. The measurement of water contact angle indicated that the IOL material has good hydrophilicity, which may lead to the biological adhesion between human lens epithelial cells and proteins. The drug-release behavior indicates that the PDA (DOX) mpc-modified IOL material, as an original drug delivery system, has long-term sustained drug release characteristics (Fig. 2d). Biocompatible elements (niobium (Nb) and silicon (Si) were introduced into a TiO_2_ matrix to change the surface chemical composition and tailor the thermophysical properties, which, in turn, leads to the generation of topographical features under specific thermal history of plasma spraying. The results indicate that the incorporation of Nb_2_O_5_ can enhance the biological performance of TiO_2_ coatings by changing the surface chemical composition and nanotopography, suggesting its potential use in the modification of biomedical TiO_2_ coatings in orthopedic applications.^32^ Stepulane *et al.* presented a polydimethylsiloxane (PDMS) surface modification strategy of antibacterial coating.^33^ Physical immobilization through the development of an interpenetrating polymer network allowed for the deposition of the microparticle coating made of cross-linked triblock copolymers (diacrylated Pluronic F127) on PDMS. A powerful antimicrobial peptide (AMP) was covalently immobilized on the surface of the produced coatings. With regard to *Staphylococcus aureus* and *Staphylococcus epidermidis*, it has a strong contact-killing antibacterial activity. Additionally, the coating's capacity to host polar, amphiphilic, and nonpolar medicines in a selective manner was evaluated, producing sustained release profiles (Fig. 3a). Xiang *et al.* synthesized a series of poly(2-phenoxyethyl methacrylate-*co*-2-phenoxyethyl acrylate-*co*-2-ethylhexyl methacrylate) (PPPE) acrylic intraocular lens (IOL) materials for “glistening-free” optimization.^34^ The 2-ethylhexyl methacrylate content in the chosen PPPE at 2% demonstrated good optical, foldable, and thermomechanical capabilities. Following gentamycin conjugation (PDA/GS), polydopamine was coated on the front side of PPPE. It reduced the thickness of the biofilm by 87% and prevented bacterial adherence by 74%. Bacterial growth was controlled in the inflammatory-mimicking conditions by acid-dependent GS release behavior. The PPPE surface remained hydrophobic as it approached the posterior capsule. The attachment of human lens epithelial cells, the adsorption of collagen IV and fibronectin, and the subsequent development of a “sealed sandwich structure” were all made possible by this (Fig. 3b).^35^ To efficiently prevent PCO, the hydrophobic surface of IOLs facilitates the inhibition of irregular migration and proliferation of human lens epithelial cells (HLECs). Therefore, an IOL material with a two-sided heterogeneous surface was needed. Photodynamic coating was introduced into the IOL surface modification. The photosensitizer chlorin e6-grafted a-cyclodextrin (a-CD-Ce6) was synthesized and self-assembled onto poly(ethylene glycol) methacrylate (PPEGMA) brushes. It established the IOL surface *via* supramolecular interactions between a-CD and polymer chains. The results indicated that this functional coating modification was effective in eliminating cells from the IOL surface when treated with light, while sustaining cytocompatibility in the absence of light (Fig. 3c).

**Table 1:** Coating strategies used for the generation of multifunctional surfaces

Reference	Methods	Substrates	Cell experiments/microorganisms	Biocompatibility/bioactivity/antibacterial activity
27	*N*-hydroxysuccinimide (NHS) esterandmaleimide functionalities, chitosan, growth factor protein (FGFr2) molecules	Tissue culture polystyrene (TCPS)	Adipose-derived stem cells (ADSCs)	Cellular spheroids exhibit an enhanced growth activity in terms of size (210% larger) and number (180% less spheroids) ADSCs cell growth activity and proliferation were enhanced
29	Fe-NP, hydrogel	Metallic stent	Human umbilical cord vein endothelial cells (HUVEC)	Promoting endothelial cell adhesion, proliferation, and migration
30	Tricalcium phosphate (β-TCP), silver–silica (Ag/SiO_2_) nanocomposite	NiTi shape memory alloy	*E. coli*, *Staphylococcus aureus* (*S. aureus*), *Saccharomyces cerevisiae* (*S. cerevisiae*)	The coating with silver doped inhibits organization of biofilm created by Gram-negative bacteria, antimicrobial activity, *E. coli* viability decreased, non-cytotoxic
31	Doxorubicin (DOX), polydopamine (PDA), 2-methacryloyloxyethyl phosphorylcholine (MPC)	Intraocular lens (IOL)	Human lens epithelial cell line (HLE B3, CRL-11421)	The cell viability on the drug-loaded coating, surfaces decreased to around 40 or 20%, favorable biocompatibility, the excellent hydrophilicity
33	Antimicrobial peptides (AMP), diacrylate pluronic F127 (DAF127) hydrogel	Polydimethylsiloxane (PDMS)	*S. Epidermidis*, *S. aureus*	Significant reduction in the surface-adhered bacteria counts, up to 99.3% reduction of *S. epidermidis*, antibacterial activity of 99.1% against *S. aureus*
34	Polydopamine, (PDA), gentamycin sulfate (GS)	Poly(2-phenoxyethyl methacrylate-*co*-2-phenoxyethyl acrylate-*co*-2-ethylhexyl methacrylate) (PPPE)	Human lens epithelial cells (HLECs) *S. aureus*, *P. aeruginosa*	Antibacterial adhesion rates of 63.8%, 73.7% against *S. aureus* and *P. aeruginosa* respectively, biofilm formation was effectively inhibited, cell viabilities of HLECs were above 90%
35	α-Cyclodextrin (α-CD), chlorin e6, poly(poly(ethylene glycol)methacrylate) (PPEGMA)	IOL	HLE B3, CRL-11421	Excellent biocompatibility, increased cell adhesion and proliferation

The use of grafting, assembly and other methods to improve hydrophilicity requires special equipment or complex preparation conditions, which has limitations in practical applications. However, the preparation process of surface coating modification technology is simple, and the coating thickness and composition ratio are controllable. The surface properties of biomaterials functionalized with surface-modified layers of surface coatings have been improved, such as good blood compatibility, enhanced cell behaviour, expansibility, good hydrophilicity and extended life of biomaterials. However, it is essential to improve the stability of the surface coating, which is prone to fall off into fragments and produce toxicity to surrounding cells and tissues.

## Covalent grafting technology in surface modification processes

**Fig. 4:**
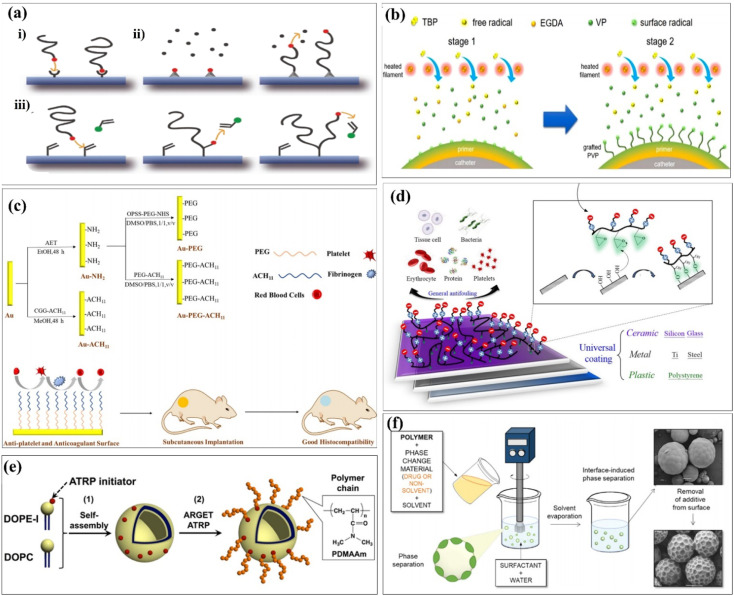
Different strategies of chemical grafting onto surfaces formed multifunctional biomaterials. (a) Schematic depiction of different strategies of chemical grafting onto surfaces: (i) grafting-to, (ii) grafting-from, and (iii) grafting-through approaches consisting of the attachment step and further chain growth. (b) (ii) illustration of the two-staged iCVD process of gPVP coating and the molecular structures of VP, EGDA, and TBP. (c) Schematic of the fabricated surface with multiple biofunctions. (d) Schematic illustration of the zwitterionic surface grafting on versatile substrates of ceramic, metal, and plastics. (e) Illustration of liposome-surface-initiated ATRP. (f) Preparation of surface-modified polymer microparticles. These are reprinted with permission from Sun *et al.* (2020, *Macromol. Rapid. Commun.*), Abdel-Rahman *et al.* (2022, *Int. J. Biol. Macromol.*), Zhao *et al.* (2019, *ACS Biomater. Sci. Eng.*), Chou *et al.* (2016, *Acta Biomater.*), Masuda *et al.* (2019, *Langmuir*), Alvarez-Paino *et al.* (2019, *ACS Appl. Mater. Interfaces*), respectively.

Chemical interactions are typical examples of establishing connections between different functional groups. It could be introduced as an important case of covalent grafting technology. Generally speaking, chemical grafting is more advantageous than physical methods because the covalent attachment of grafted chains onto the substrate surface can avoid the desorption and ensure long-term stability. Further, due to the strong, selective, specific, and convenient reactivity of polymer brushes, polymer brushes that can be chemically grafted to the surface have high adhesive strength.^36^ Chemical surface modification methods usually based on the grafting can be categorized into “grafting-to,” “grafting-from,” and “grafting-through” approaches^37^ (Fig. 4a). It was commonly used for ensuring covalent grafting of polymers.^38,39^ In the grafting-to approach, end-group-transformed polymer chains reacted with the functional groups of substrates, which finally form grafted polymer chains. In the grafting-from approaches, polymer chains propagated from surface-attached initiators and polymer chains completely propagated from surface-attached double bonds.^40^ The characteristic of the graft copolymer is controlled by the nature of grafted chains, including number, length and molecular structure of grafted chains. So far, extensive research has been conducted to explore the influences of these parameters on the grafting percentage and the characteristics of grafted materials.^41^ Covalent grafting offers the strongest link work between biomaterials and its coating candidates, and it produces a more durable interface as much as possible. A summary of the common surface modification strategies is given in Table 2.

**Table 2:** Covalent grafting strategies used for the generation of multifunctional surfaces

Reference	Surface functional groups	Substrates	Cell experiments	Biocompatibility/bioactivity
45	Grafted polyvinylpyrrolidone (PVP)	Polyvinyl chloride (PVC) medical catheters	*E. coli*	Fouling resistance of 99.98% against *E. coli*
				Good compatibility
				High surface hydrophilicity
58	Poly(ethylene glycol) (PEG), antithrombotic peptide ACH11	Au substrates	Platelet	Excellent hydrophilic property
				Strong antiprotein and antiplatelet attachment ability
				Excellent hemocompatibility
				Inhibiting granulation formation and fibrous capsule
59	Ploy(glycidyl methacrylate-*co*-sulfobetaine methacrylate) (poly(GMA-*co*-SBMA))	Silicon, glass, titanium, stainless steel, and polymers	Human blood cells, fibroblast cells, *E. coli*	Reduced by 89.3%, 73.7%, 76.5%, 77.9%, and 49.6%, for platelet adhesion and by 96.1%, 96.2%, 83.7%, 88.1%, and 87% for erythrocyte adhesion with the surfaces respectively
				A high reduction in human fibroblast (HT1080) adhesion of 96.3%, 96.5%, 91.3%, 86.3%, and 57.8% respectively
				The high reduction in bacterial attachment of 88.5%, 79.7%, 86.9%, 90.8%, and 51.4% respectively
60	*N*,*N*-Dimethylacrylamide (DMAAm)	Liposomes	N/A	Increased in surface softness
63	3-Aminopropylmethacrylamide (APMA), poly(ethylene glycol) methacrylate (PEGMA)	Poly(d,l-lactic acid) (PLA)	Human immortalized mesenchymal stem cell (hiMSCs), cardiomyocytes derived from human-induced pluripotent stem cells (hiPSC-CMs)	Non-cytotoxic to hiMSCs
				The size and number of hiMSC
				Aggregates formed are primarily influenced by the surface chemistry of the incorporated microparticles

Naim *et al.* first reported that the chemical medication of chitin by grafting with polystyrene can use ammonium persulfate (APS) as an initiator.^42^ Yu *et al.* prepared amphiphilic linoleic acid (LA) and poly(*b*-malic acid) (PMLA) double-grafted chitosans with various grafting degrees of linoleic acid (LA), which was modified with folate (FA) and poly(β-malic acid) PMLA. It was double-grafted by the chitosan to probe the optimized hydrophobicity and hydrophilicity in a co-delivery system. This co-delivery system showed significant enhancement in *in vitro* and *in vivo* antitumor efficiency while compared with single administration.^43^ Chen *et al.* used functionalized chitosan/hydroxyapatite (CS/HA) biomimetic composite scaffolds for the controlled delivery of BMP2-derived peptides (P24) through chemically grafting chitosan-4-thiobutylamidne (CS-TBA). Second, the resulting CS P24 was then combined with HA to prepare CS-P24/HA scaffolds. The effect of the CS-P24/HA scaffold on bone regeneration was evaluated, along with the underlying biological mechanisms. Finally, CS-P24/HA was superior to CS/HA in promotion of bone regeneration *in vivo*. This study highlights the enormous potential of using the CS-P24/HA scaffold for bone tissue engineering works.^44^ Since an ultralow fouling surface is essential for good biocompatibility, Sun *et al.* attempted to further enhance the surface functionality using a novel solvent-free graft-from method. The grafting was implemented by first depositing a cross-linked polyvinylpyrrolidone (PVP) prime layer *via* initiated chemical vapor deposition (iCVD), followed by *in situ* polymerization on the top position without disrupting the vacuum. The propagating chains on the prime coating surface served as the initiating sites for the subsequent grafting of PVP. The resulting coating thus consisted of a cross-linked prime layer and a top-grafted PVP layer.^45^ Liposomes are self-assembled vesicles of amphiphilic lipid molecules, and they are treated as cells, model of cells, or tools for drug delivery systems. Modifications of polymer chains on the surfaces of liposomes are typically installed using a “grafting to” approach,^47^ as shown in Fig. 4b. Abdel-Rahman *et al.* prepared a 3D hybrid scaffold based on a collagen-*grafted*-chitosan–glucan fiber (CO-*g*-CGF–HBS) by a freeze-drying technique. The swelling percentage, hydrolytic stability, and modulus of elasticity of HBS were enhanced after the chemical modification of CO with CGF. The chemical modification of CO with different ratios of CGF significantly improved the physicochemical and antibacterial properties of HBS.^46^

The initial attachment of bacteria to the surface of implanted medical devices is the first stage in biofilm formation. Thus, the inhibition of bacterial colonization using antibacterial coatings, particularly during the most susceptible first 6 h period after implantation, is a fundamental strategy to prevent biofilm formation.^48–51^ To achieve ultralow fouling surfaces, hydrophilic polymer coatings such as polyethylene glycol (PEG)^52–55^ and zwitterionic polymers^56,57^ have been extensively explored. The grafted chains generate a bound hydration layer that prevents the contact between foulants and the surface and induces a “cushion effect” repelling the approaching foulants.^55^ Zhao *et al.* developed a PEG- and ACH_11_-functionalized surface.^58^ Hydrophilic poly(ethylene glycol) (PEG) and antithrombotic peptide ACH_11_ were co-immobilized onto Au to develop a multifunctional surface with remarkable hemocompatibility, protein adsorption, antiplatelet aggregation, anticoagulant properties, and good histocompatibility (Fig. 4c). Chou *et al.* reported that a facile, effective and economic grafting-to method, which forms a stable chemisorption coating on versatile surfaces, was developed by the original design in the zwitterionic copolymer formulation of poly(glycidyl methacrylate-*co*-sulfobetaine methacrylate) (poly(GMA-*co*-SBMA)).^59^ The epoxide functional groups of GMA segments could provide strong reactivity with nucleophiles *via* the ring-opening reaction. Thus, poly(GMA-*co*-SBMA) is capable of forming covalent bonding with versatile surfaces including polymer, ceramic, and metal substrates with hydroxyl groups after surface pre-treatment using UV and ozone, as shown in Fig. 4d. Masuda *et al.* hypothesized that ARGET ATRP could be used to modify a liposome surface by the grafting-from method.^60^ The molecular weight of the grafted polymer chain was systematically controlled by changing the monomer concentrations in the “grafting from” polymerization, as shown in Fig. 4e. Zhou *et al.* reported that ethyl ketones (AAEK) were covalently grafted onto cellulose films (CF) *via* a copper-catalyzed azide–alkyne 1,3-dipolar cycloaddition click reaction.^61^ The purpose of this study is to explore the effectiveness of surface-decorated aryl(β-amino) AAEK, a promised enzyme A (SrtA) inhibitor of *Staphylococcus aureus*, to improve the anti-adhesion ability of biomaterials. Du *et al.* synthesized and grafted AACA onto the surface of PIB by plasma pre-treatment and UV-induced graft polymerization.^62^ The hydrophilicity and hemocompatibility of PIB were largely improved by surface modification of aminocaproic acid (AACA), which were confirmed by water contact angle and platelet adhesion, respectively. Alvarez-Paino *et al.* reported a new approach for surface functionalization of poly(lactic acid) (PLA) microparticles that allows the decoration of the outer shell of the polyesters with additional functionalized poly(poly(ethylene glycol) methacrylate) and poly[*N*-(3-aminopropyl)methacrylamide] brushes, chosen for their potential abilities to mediate cell adhesion,^63^ as shown in Fig. 4f.

In summary, the surface grafting modification technology significantly enhances the surface properties of biomaterials such as cell adhesion, hydrophilicity, biocompatibility, and stain resistance, while maintaining the same characteristics of the substrate material. Surface coating has the disadvantage of instability and easy shedding, while surface grafting can enhance its stability in the physiological environment, and the grafted chain can prevent dirt from contacting the surface of biomaterials. Obviously, the traditional organic solvent-based polymer grafting method is lengthy and complicated, which is easy to contaminate the material surface. The solvent-free grafting method can obtain better surface properties to a certain extent. Surface grafting requires less energy and is more environmentally friendly than plasma surface modification techniques. The functionalization of spherical particles cannot be controlled uniformly by plasma treatment, and the morphology of the underlying material can also be altered. However, the limitation of surface grafting may lead to inefficient antifouling properties, and the precise control of the synthetic process on the surface of biomaterials remains flawed and needs to be improved.

## Self-assembled monolayers as a surface modification strategy

Self-assembled monolayers (SAMs) are nano-thick ordered molecular coatings formed by molecular components adsorbed from solution or gas phase to solid surface and finally arranged in a regular manner.^64^ The molecules from SAMs are well bound to the surface of the substrate material and have specific functional groups. A summary of the common surface modification strategies is given in Table 3.

**Table 3:** Self-assembled monolayer strategies used for the generation of multifunctional surfaces

Reference	Surface functional groups	Substrates	Cell experiments	Biocompatibility/bioactivity
68	Amine (NH_2_), octyl (CH_3_)	Ti_6_Al_4_V	Mouse fibroblast L929 cells, *Staphylococcus aureus*, *Escherichia coli*	Hydrophobic octyl surfaces (1035 ± 38 ng cm^−2^) showed the maximum adsorption of bovine serum albumin (BSA)
				Hydrophilic COOH surfaces (647 ± 38 ng cm^−2^) showed the maximum adsorption of fibronectin (FN)
				Hybrid surfaces showed the maximum cell adhesion (%) and proliferation, larger nuclei area and the least cell circularity
				Cells exhibited higher proliferation rate on all the modified surfaces as compared to unmodified Ti_6_Al_4_V, suggesting good cytocompatibility
69	Silanization	Silicon wafers	Serum, saliva	The modified materials that span a broad range of physicochemical properties, from hydrophilic to hydrophobic surfaces (water contact angles from 15° to 115°), negative to positive surface charge (zeta potentials from −120 to +40 mV at physiologic pH)
				The chemical surface functionalities exerted a substantial effect on the total amounts of proteins adsorbed
71	Different chemical groups (–OH, –OEG, –COOH, –NH_2_, and –PO_3_H_2_)	Gold slides	Recombinant mouse osteopontin (OPN), mouse bonemarrow mesenchymal stem cells (mBMSCs)	The amount of adsorbed OPN was highest on SAMs-NH_2_ (89.01 ± 13.62 ng cm^−2^) and lowest on SAMs-OEG (3.39 ± 0.63 ng cm^−2^)
				Cells on SAMs-COOH, SAMs-NH_2_, and SAMs-PO_3_H_2_ with pre-adsorbed OPN showed larger spreading, better viabilities, and higher expression levels of *α*_v_/*β*_3_ genes
				OPN on SAMs-COOH, SAMs-NH_2_, and SAMs-PO_3_H_2_ exhibited higher bioactivity
77	Thioctic acid-functionalized dendritic polyglycerol sulfate (dPGS)	Gold-coated sensors	Blood proteins albumin (Alb) and fibrinogen (Fib)	Compared to non-sulfated dPG, dPGS showed enhanced protein adsorption driven by ionic interactions and enhanced cellular uptake *via* the formed protein corona
				The formation of densely packed protein layers in case of Fib and a more loosely packed protein layer in case of Alb
80	Self-assembled monolayer of phosphonates (SAMPs)	Silicon dioxide (SiO_2_)	Chondrocytes	Chondrocytes attach to the modified surface, without substantial changes in gene expression SAMPs modification to SiO_2_ increased chondrocyte adhesion by 3× after 4 h and 4.5× after 24 h
81	[(3-Aminopropyl)triethoxysilae (APTES), and octadecyltrimethoxysilane (OTS)], amino acid (histidine and leucine)-conjugated	Poly(dimethylsiloxane) (PDMS)	Induced pluripotent stem cells (iPSCs)	Modified surfaces were found to be hydrophilic
				PDMS surface chemical properties were enhanced for the differentiation of iPSCs into cardiomyocytes
				All SAM-modified surfaces increased the number of viable iPSCs, when compared to native PDMS
84	ω-(Ethylene glycol)_2–4_- and ω-(GRGDS)-, α-benzamidinobolaamphiphiles	Gold	MC3T3-E1 cells	Modified surfaces can be used to reverse cell adhesion in a noninvasive manner
				A versatile tool to study and control cell adhesion and differentiation
85	Antimicrobial peptides (AMPs), elastin-like recombinamers (ELRs)	Gold sputtered cover glasses	*S. aureus* ATCC 25923, *S. epidermidis* ATCC 35984	The multifunctional SAMs exhibit protein anti-fouling activity
				Strong anti-biofilm activity and cytocompatibility of these coatings was demonstrated
86	Chitosan layer	Polyetheretherketone (PEEK)	MC3T3-E1, Gram-negative *P. gingivalis*, Gram-positive *S. mutans*	The inclusion of chitosan on the surface of PEEK-CS increased fibronectin adherence, enhancing the adhesion, proliferation, and differentiation of MC3T3-E1 subclone 14 cells substantially
				Modified PEEK surfaces demonstrated decreased adhesion force to *P. gingivalis*, and less initial bacterial adhesion to *P. gingivalis* and *S. mutans*

**Fig. 5:**
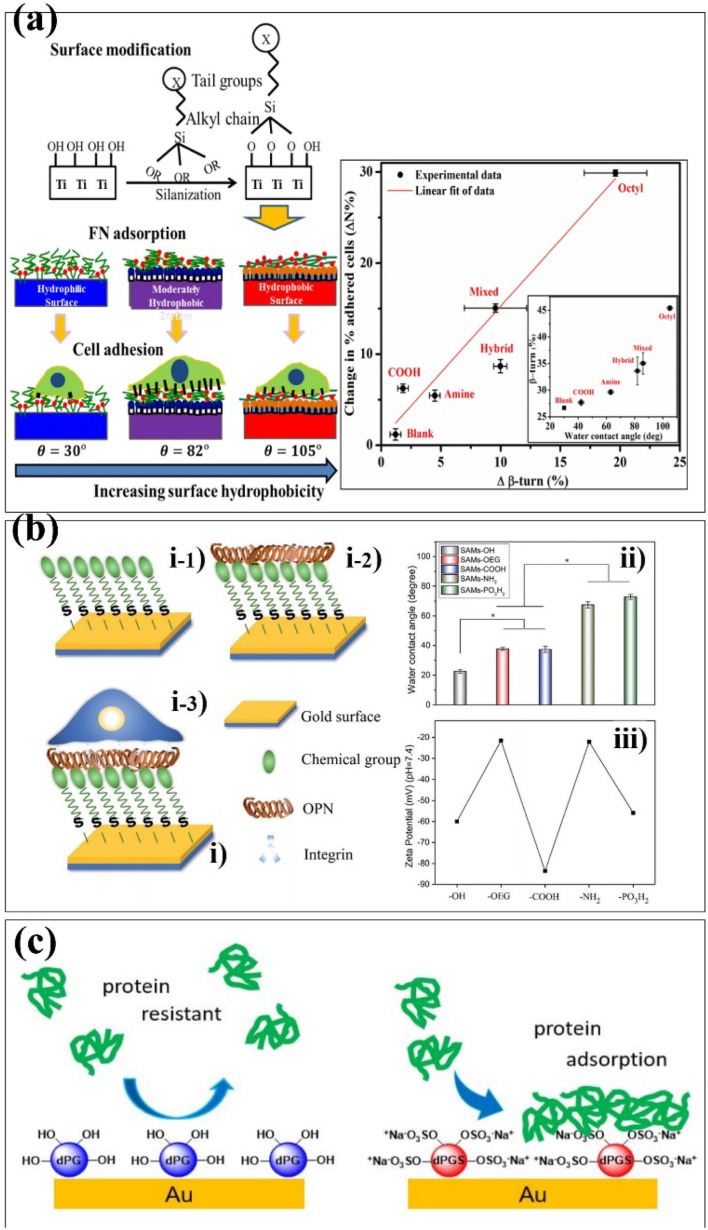
Schematic depiction of different self-assembled strategies onto biomaterials surfaces. (a) Schematic of silanization on the Ti surface was used to study fibronectin and cell adsorption. (b) (i) Preparing SAMs with different terminal chemical groups for OPN adsorption and cell adhesion studies: (i-1) chemical groups (–OH, –OEG, –COOH, –NH_2_, and –PO_3_H_2_) were first self-assembled on a gold slide; then (i-2) OPN was adsorbed on the SAMs; and (i-3) MSCs were finally seeded and adhered on these SAMs. (ii) Surface wettability and (iii) surface zeta potential data from the obtained SAMs. The stars in (ii) indicate significant difference (*p* < 0.05). (c) Schematic of thioctic acid-functionalized dPG and dPGs assembled on gold surfaces was used to study protein adsorption. These are reprinted with permission from Hasan *et al.* (2018, *Langmuir*), Chen *et al.* (2021, *RSC Adv.*), Stöbener *et al.* (2018, *Langmuir*), respectively.

The SAM surface modification technology has been widely used in protein adhesion, cell adhesion, antibacterial and antifouling. The properties of biomaterials are mainly determined by the proteins adsorbed on their surfaces.^65,66^ These proteins play a role in regulating cell adhesion, migration, proliferation and differentiation.^67^ Therefore, it is essential to regulate the adhesion of proteins on the surface of biomaterials. Hasan *et al.* investigated the effect of SAM-functionalized biomaterials on protein adsorption.^68^ The ability of Ti_6_Al_4_V to adsorb proteins and adhere to cells was tested by forming a hydrophilic, hydrophobic, and moderately hydrophobic monolayer on its surface. Similar as surface coating works, researchers controlled the surface properties of Ti_6_Al_4_V using a technique called silanization, which forms a covalent bond between the surface molecules linkages. It makes the biomaterials more stable and robust in performance (Fig. 5a). Lehnfeld *et al.* developed an *in vitro* model system based on silica surface in order to investigate the effect of adsorbed protein layers on the chemical modification of biomaterials.^69^ It was modified by seven silanized SAMs. The results show that the chemical surface function of biomaterials greatly affects the total amounts of proteins adsorbed. Osteopontin (OPN) can mediate the cell behaviour of materials.^70^ Chen *et al.* reported SAMs with different surface chemistries to investigate the behaviour of OPN on these SAMs.^71^ The results indicated that the adsorption capacity of SAMs-NH_2_ to OPN was stronger and the protein content was the highest. Meanwhile, *in vitro* cell experiments indicated that SAMs-COOH, SAMs-NH_2_ and SAMs-PO_3_H_2_ adsorbed with OPN can promote cell behaviour. It also proved that the SAM adsorbed with OPN has higher bioactivity, providing a new idea for the surface modification of biomaterials (Fig. 5b). Biomaterials often come into contact with blood when implanted in the body and it is essential to avoid the adsorption of non-specific proteins on the surface.^72–74^ Previous studies have shown that dendritic polyglycerin (dPG) protects biomaterial surfaces from non-specific protein adsorption and effectively reduces the adhesion of undiluted serum proteins.^75^ In addition, a polyvalent form of dendritic polyglycerol sulfates (dPGS), which can effectively inhibit and bind to inflammation *in vivo*, was introduced.^76^ Stöbener *et al.* obtained a stable dPGS self-assembled monolayer on a gold substrate surface, which was acid-functionalized thioctic.^77^ Compared with unsulfated dPGS, there are many negatively charged sulfate groups on the surface of dPGS, which enhance the adsorption of proteins under ionic interactions and lead to the partial rearrangement of the protein structures (Fig. 5c).

**Fig. 6:**
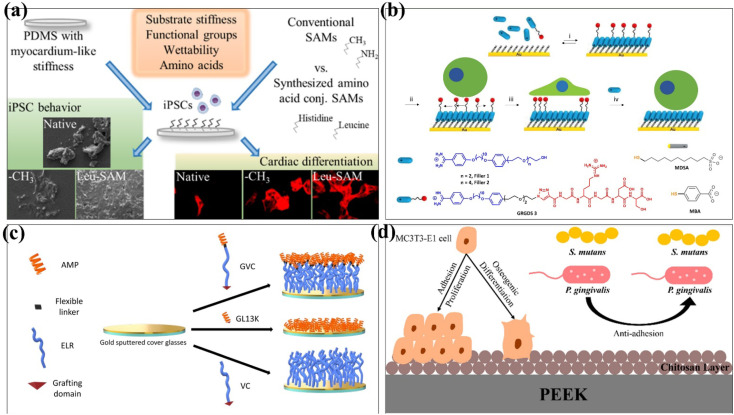
Schematic depiction of different self-assembled strategies onto biomaterials surfaces. (a) Scheme illustration of the preparation of PDMS substrates having the similar stiffness as a healthy heart tissue and a well-defined surface chemistry obtained by conventional [(3-aminopropyl) triethoxysilane (APTES) and octadecyltrimethoxysilane (OTS)] and amino acid (histidine and leucine)-conjugated SAMs. (b) Scheme illustration of the supramolecular-based approach to prepare reversible self-assembled monolayers (rSAMs) with tunable lateral mobility and dynamic control over surface composition to regulate cell adhesion behaviour. (c) Schematic of the modular composition of the AM-ELR and production of the AMP/ELR/AMELR self-assembled monolayers (SAMs) on gold surfaces. (d) Schematic illustration of dual-functional polyetheretherketone surface with an enhanced osteogenic capability and an antibacterial adhesion property *in vitro* by chitosan modification. These are reprinted with permission from Öztürk-Öncel *et al.* (2021, *ACS Biomater. Sci. Eng.*), Yeung *et al.* (2022, *ACS Appl. Mater. Interfaces*), Acosta *et al.* (2019, *ACS Biomater. Sci. Eng.*), Qiu *et al.* (2022, *Langmuir*), respectively.

Cells attached to the surface of biomaterials can perceive and respond to chemical and physical surface features.^78,79^ Therefore, the control of cell adhesion behaviour should be considered in the design of biomaterials to influence cell migration, proliferation and differentiation. Donnelly *et al.* investigated the effect of the self-assembled monolayer of phosphonates (SAMPs) on chondrocyte adhesion on silica (SiO_2_) and polyvinyl alcohol (PVA) materials.^80^ The experimental results showed that chondrocytes attached to SAMP–SiO_2_ increased 3 times after 4 hours and 4.5 times after 24 hours. At the same time, the modified PVA material increased the number of chondrocytes by at least 31 times in the same time. In conclusion, the SAMP surface modification technology can improve chondrocyte adhesion and diffusion on the material without altering gene expression. Functional groups on the SAMs have been found to enhance cell interactions with biomaterials and induce stem cell differentiation.^81^ For example, amino (–NH_3_)-conjugated SAMs promote osteogenic differentiation,^82^ while methyl (–CH_3_) supports chondrogenesis in stem cells.^83^ Öztürk-Öncel *et al.* modified different functional groups on the surface of poly(dimethylsiloxane) (PDMS) substrate using amino acid-conjugated SAMs.^81^ Amino acid-conjugated SAM-modified materials improved induced pluripotent stem cell (iPSCs) behaviour, and these functional groups with different surface functions had synergistic effects on iPSCs attachment, viability, and cardiomyocyte differentiation (Fig. 6a). Yeung *et al.* reported a reversible self-assembled monolayer (rSAMs) that achieves the dynamic control of surface composition and adjustable lateral fluidity by adding inert filler amphiphiles to the tripeptide RGD-functionalized rSAMs, so as to achieve the purpose of regulating cell adhesion behaviour^84^ (Fig. 6b).

SAM-modified biomaterials have also been widely used in antibacterial and antifouling fields. In 2019, Acosta *et al.* used antimicrobial peptides (AMPs) and elastin-like recombinamers (ELRs) to design self-assembled monolayers.^85^ The antibacterial properties of GL13K peptide and the low pollution activity of ELRs were combined and fixed on the surface of gold substrates by covalent grafting. The recombinant AMP/peptide SAM was evaluated to provide anti-biofilm properties against *Staphylococcus epidermidis* and *Staphylococcus aureus* (Fig. 6c). Qiu *et al.* used a self-assembly method to covalently graft the chitosan layer onto the surface of PEEK by hydroxylation, introducing amino groups and glutaraldehyde crosslinking.^86^ The method changes the surface morphology as PEEK increased the surface roughness and decreased the contact angle. The rough PEEK-CS surfaces are more likely to osteogenic than smooth PEEK-CS surfaces, which is attributed to the higher osteogenic activity of rough implant surfaces and in favour of cell adhesion, cell proliferation, and calcium nodule deposition.^87^ The adhesion of bacteria on PEEK-CS to *Porphyromonas gingivalis* and *Streptococcus mutans* was decreased. Chitosan modification significantly improved the osteogenic ability and antibacterial adhesion of polyether ether ketone *in vitro* (Fig. 6d).

Different from surface coating modification, self-assembled monolayers can form coatings on various metal surfaces with high flexibility. At the same time, some SAMs also exhibit good thermal and hydrolytic stability. SAMs are controllable for cell adhesion and do not change the overall mechanical properties or surface roughness of the biomaterials, resulting in better biocompatibility of the materials. The different functional groups of the self-assembled monolayer make the interaction between cells and biomaterials different. The fabrication of self-assembled monolayers is simple and does not require expensive and sophisticated equipment. Although self-assembled monolayer-modified biomaterials have shown good antibacterial and osteogenic properties *in vitro*, *in vivo* antibacterial experiments are still insufficient. The long-term stability of functionalized biomaterials needs to be verified, especially for orthopedic and dental applications.

## Plasma strategies in surface modification processes

Plasma is a substance that exists in the fourth state, which can be regarded as a collection of electrons, single- or multiple charged positive and negative ions accompanied by neutral atoms, excited particles, electromagnetic radiation, molecules, and molecular fragments which are in charge balance.^88,89^ In most experimental situations, the plasma is generated by discharge.^88^ It can generate the energy and produce plasma by applying electric and magnetic fields or even nuclear reactions.^90^ This method has the advantage of only changing the surface properties of the material, such as surface chemistry, roughness, surface charge, and also enhancing the biocompatibility of the material, while the bulk properties of the material remain unchanged.^90,91^ Gas plasmas can be generated under both atmospheric pressure and low-pressure conditions.^92^ A large number of gases can be used in plasma, including argon, nitrogen, and argon–oxygen mixture, which are used to generate functional groups or free radicals on the surface of materials. These free radicals can improve the adhesion of the surface of biomaterials or graft other polymers on it to induce surface hydrophilicity.^93,94^ Conversely, conventional gas plasma treatments and plasma immersion ion implantation can be conformal modified around the material, allowing treatment of 3D structures with high surface area and volume ratios with a wide range of internal porosity.^95^ According to the interaction between the plasma and the surface of biomaterials, it can be divided into three categories: plasma polymerization, plasma treatment, and plasma graft etching.^94^ Plasma surface modification techniques can be used directly or in combination with other surface modification techniques to achieve the purpose of enhancing the surface properties of biomaterials.^93^ This section reviews the gas plasma strategy and plasma immersion ion implantation strategy in the literature in recent years, discussing and summarizing several studies on plasma treatment of different biomaterials to illustrate the importance of plasma technology in the surface modification of biomaterials. A summary of the common surface modification strategies is given in Table 4.

**Table 4:** Plasma strategies used for the generation of multifunctional surfaces

Reference	Methods	Substrate	Cell experiments	Biocompatibility/bioactivity
96	Oxygen plasma	Quartz	Bone marrow stromal cells (BMSCs) MC3T3-E1 cells	Cell density increased from 34 ± 1 cells per mm^2^ to 78 ± 3 cells per mm^2^, obvious graded distribution, oriented migration
99	Argon plasma	Poly(lactic-*co*-glycolic acid) (PLGA)	N/A	Water contact angle dropped from 70° to 42°
100	Argon and argon–oxygen plasma	Nanofibers	Fibroblast cells	Further increase in the number of fibroblast cells (145 ± 9)
101	CH_4_, C_2_F_6_, oxygen	General-purpose polystyrene (GPPS)	*S. aureus*, *E. coli*	Antibacterial activity *R* = 4.1146, has antibacterial effect
102	Diethyl phosphite (DEP)	Titanium (Ti)	*C. Albicans* and *S. aureus* L929 fibroblast cells	72 h of antifungal assay, 13 cfu mL^−1^ of *S. aureus* and no live *C. albicans* was observed, increased cell viability of L929 fibroblast cells (93%)
105	Nitrogen ion (PIII)	Silk films	Bovine arterial endothelial cells (BAECs)	Cell adhesion on PIII treated silk films was 2.5 s fold higher than on untreated silk films, PIII-treated silk supported significantly higher cell proliferation
106	C, nitrogen (PIII)	Zirconia	BMSCs	The number of cells on zirconia disks was significantly increased, the number of cells attached on surface was highest among the four groups (*P* < 0.05), C and N2-PIII promoted cell proliferation activity
107	Nitrogen ion (PIII)	Silk films	Plasma proteins	Recombinantly expressed domain V of the human the rate and the amount of basement membrane proteoglycan perlecan (rDV) binding were greater on PIII silk, rDV bioactivity was retained following covalent immobilization on silk, rDV-functionalized PIII silk inhibits thrombogenic activity in whole blood systems

**Fig. 7:**
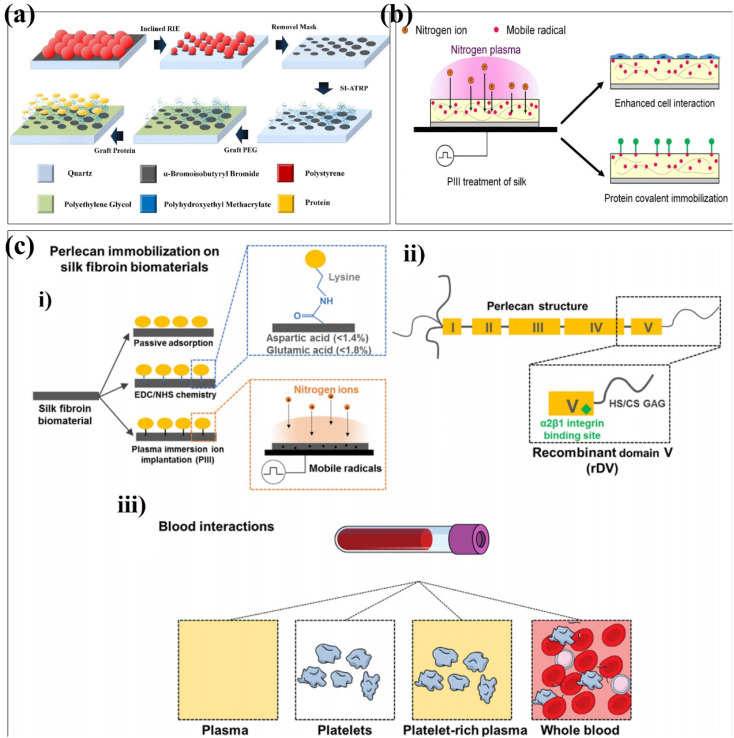
Schematic depiction of plasma strategies onto surface-formed multifunctional biomaterials. (a) Schematic illustration of the fabricating process of graded protein/PEG nanopattern arrays. (b) Schematic of the fabrication process of surface modification using plasma immersion ion implantation (PIII). (c) Schematic of silk biofunctionalization with recombinantly expressed domain V (rDV) of human perlecan for blood contacting applications. (i) Schematic of the silk film biofunctionalization with rDV *via* passive adsorption (silk), carbodiimide chemistry (EDC/NHS silk) or nitrogen plasma immersion ion implantation (PIII silk). EDC/NHS-based protein immobilization occurs *via* formation of an amide bond between a carboxylic acid and a primary amine, typically occurring between aspartic/glutamic acid on silk and lysine on rDV. PIII-based immobilization relies on diffusion of mobile radicals from the PIII-treated silk surface to react with rDV. (ii) Schematic of the main structural and functional features of perlecan domain V expressed recombinantly in HEK-293 cells. rDV is a proteoglycan consisting of an 80 kDa protein core decorated with either a heparan sulfate (HS) or a chondroitin sulfate (CS) glycosaminoglycan (GAG) chain and has an α2β1 cell integrin binding site located at the C-terminus of the proteoglycan. (iii) Schematic of blood contacting assays used in this study with an increasing amount of blood components and complexity of interaction with the exposed surface. These are reprinted with permission from Xue *et al.* (2019, *ACS Appl. Mater. Interfaces*), Kondyurin *et al.* (2018, *ACS Appl. Mater. Interfaces*), Lau *et al.* (2021, *Acta Biomater.*), respectively.

Xue *et al.* grafted a layer of α-bromoisobutyryl bromide (initiator) on the surface of quartz substrates treated with oxygen plasma.^96^ Subsequently, to protect the initiator from oxygen plasma corrosion in inclined reactive ion etching (RIE), a polymer film was coated as a protective layer.^97,98^ The geometric gradient was introduced into the prepared polystyrene (PS) microspheres array by inclining RIE. Since the polymer film is etched away, the gaps between the initiators are filled with silica hydroxyl groups. Gradient polyhydroxyethyl methacrylate (PHEMA)/polyethylene glycol nanopatterned arrays were fabricated from gradient initiator/PEG nanopatterned arrays by surface-initiated atom-transfer radical polymerization (SI-ATAP) on quartz substrates. Finally, the protein was covalently immobilized on PHEMA nanodots, resulting in graded and ordered gradient protein/PEG nanopattern arrays (Fig. 7a). Gradient biomaterials based on plasma preparation can affect three basic behaviours of cells: adhesive density, polarization and migration. Phat *et al.* performed the surface modification of polylactic acid–glycolic acid (PLGA), collagen, and PLGA-collagen using a PDC-001 Expanded Plasma Cleaner.^99^ The contact angles of water and diiodomethane on PLGA films treated by argon plasma were significantly decreased (*p* < 0.05), but had little effects on the thermal degradation of collagen and PLGA-collagen at high temperatures. The results of the bicinchoninic acid assay showed that the argon-plasma treated scaffolds released less collagen to PBS++ (*p* < 0.05). Mozaffari *et al.* used a PF-200 plasma DBD device to plasmonic electrospun nanofiber scaffolds with argon and an argon–oxygen mixture.^100^ The surface of the untreated nanofibers was smooth, while the surface roughness of the nanofibers treated with argon and argon oxygen plasma was greatly increased, which was related to the bombardment of high-energy particles when the samples were treated with plasma. At the same time, the argon–oxygen plasma-treated nanofibers exhibited a high degree of hydrophilicity, and the water droplets were completely absorbed into the scaffold, which was related to the introduction of polar groups. In contrast to air plasma modification, Onodera *et al.* formed fluorine-containing diamond-like carbon (DLC) films on a General-Purpose Polystyrene (GPPS) substrate using CH_4_ and C_2_F_6_ as gas sources.^101^ Subsequently, the film was subjected to atmospheric plasma treatment with oxygen to improve the biocompatibility of the film. The film has antibacterial properties, and fluorine has an inhibitory effect on glucose metabolism for bacterial energy production. Physical changes have also occurred on the film surface including increased hydrophilicity and decreased surface wettability to achieve the purpose of antibacterial activity. Kaleli-Can *et al.* used the plasma polymerization technique for surface modification with titanium using diethyl phosphite (DEP) as the gas source in an RF/low-pressure plasma device under 0.15 mbar.^102^ The water contact angle of the DSP-coated Ti is significantly smaller, and the surface energy is about two times higher than that of the unmodified Ti surface. The surface roughness was increased, and it had an inhibitory effect on *C. albicans*, which showed good antibacterial activity. Amphotropic plasmonic polymer prepared from DEP can effectively prevent biofilm formation on the titanium surface.

Ion implantation is the process by which positively charged ions in plasma are accelerated into the surface of a material.^103^ Generally, the energy of the accelerated ion is much higher than the bond energy of the surface layer of the modified material, resulting in bond breaking.^104^ Kondyurin *et al.* reported the silk fibroin biomaterials using plasma immersion ion implantation (Fig. 7b).^105^ PIII transforms the surface layer of the biomaterial into a compact carbon-rich structure with no significant effect on its roughness. Due to the presence of free radicals during PIII treatment, the surface exposed to the atmosphere undergoes oxidation. PIII enhances silk interactions with proteins and cells. This provides a research direction for the application of silk fibroin biomaterials in tissue engineering and regenerative medicine in the future. It has been found that the biological activity can be improved by building functional groups. Guo *et al.* randomly divided the zirconia into four groups and used the corresponding parameters to modify the zirconia surface using plasma immersion ion implantation.^106^ The surface roughness of the material was unchanged, the nitrogen-containing functional groups were introduced, and the stability of zirconia was not affected. *In vitro* data showed that PIII-treated zirconia promoted the adhesion, proliferation, and osteogenic differentiation of bone marrow-derived mesenchymal stem cells (BMSCs). Lau *et al.* used the PIII treatment technique to covalently immobilize recombinantly expressed domain V (rDV) on silk biomaterials, independent of immobilized proteins modified by specific amino acids in the silk protein chain.^107^ The silk biomaterials biofunctionalized with rDV using PIII treatment show blood compatibility in terms of platelets (Fig. 7c). Different from plasma immersion ion implantation, researchers have also proposed ion implantation surface modification to improve properties such as biocompatibility, corrosion resistance and antibacterial properties.

In short, plasma-based surface modification techniques have great potential for biomaterial implants. Surface coating, self-assembling monolayers and ion implantation are limited to certain biological material chemistry or surface pretreatment. These chemical processes are time-consuming and complex, and require the addition of organic reagents, which may damage the implant. However, plasma surface modification technology has the advantages of short reaction time, environmental safety, and the ability to change the surface properties of the material without affecting the overall properties of the material. The interaction between plasma and some biomaterials can enhance their biocompatibility with biological cells, which can be used in tissue engineering. In addition, the films prepared by plasma surface modification have the characteristics of thermal stability, chemical inertness, and enhanced toughness. Plasma surface modification can improve the surface wettability and cell attachment of biomaterials, and also improve their osteogenic and antibacterial properties. However, during the plasma process, the choice of the precursor can affect the properties of the biomaterial, leading to results that differ from the desirable properties. If the antibacterial layer on the surface of biomaterials lacks the killing and release mechanism, its antibacterial function will be weakened or even disappeared.

## Other surface modification strategies

Appeared in healing of bone engineering, it lacks osteogenic activity of the implanted fixation material and the infection of bacteria.^108,109^ It is an important topic to pursue osteogenic activity and antibacterial properties. The previous reports have found that active ionic components play an important role in bone formation, development and repair, and that some metal ions can act as antibacterial agents.^110^ Yang *et al.* prepared a protective coating containing Zn and Sr ions by a one-pot hydrothermal method.^111^ After surface modification, a cluster of crystalline structures was formed on the surface of magnesium alloys. At the same time, the corrosion resistance of the magnesium alloy was significantly improved, and the cells grew well on the surface. The combined use of Zn and Sr ions promoted the osteogenic differentiation of the cells with antibacterial effects. Kazimierczak *et al.* have synthesized magnesium (HA-Mg) and (HA-Zn) ion-substituted nano-hydroxyapatite (HA) synthesized to prepare a more biocompatible chitosan-agarose-hydroxyapatite (HA) scaffold (chit/aga/HA).^112^ These two above-mentioned metal ions have been found to have synergistic effects on the biological response in cell tests. The addition of Mg^2+^ to this biomaterial structure can promote osteoblast spreading, promote cell proliferation on the scaffold surface, and promote osteocalcin production by mesenchymal stem cells. Moreover, the addition of Zn^2+^ can promote the production of type I collagen by MSCs and extracellular matrix calcification. It has been found that the nanostructure on the surface of biomaterials has an important effect on cell activity and tissue formation.^113^ Wen *et al.* added polydo and the physical properties were not changed. Shuai *et al.* reduced silver nanoparticles on carbon nanotubes (CNTs) *in situ*.^114^ The CNT@Ag powder was then prepared by laser powder bed fusion (LPBF) to prepare the Zn-CNTs@Ag scaffold implant, which had an orthogonal porous structure that facilitates cell migration, tissue formation, and nutrient transportation. The scaffold exhibited favourable antibacterial activity and biocompatibility.

## Conclusions and future prospects

As implanted in the human body, biomaterials have been widely used in the biological applications and clinical treatment. Especially in microbial infection, it is not easy to evitable in surgical implantation and resulted inflammation, biofilm harmful. Therefore, the surface modification could change the physical, chemical and biological properties of implant materials in order to improve the biocompatibility, antibacterial and antifouling properties. In this review, the main surface modification strategies and methods are commonly illustrated. Among this, it is an effective way to enhance the antibacterial activity and reduce the adhesion of non-specific proteins and bacteria *via* surface modification of medical biomaterials. Towards hydrophilic contact angle, surface roughness, and surface functional groups, they ensured target material acceptable and biocompatible. Although the existing surface modification results are satisfactory, there are still many deficiencies that need further investigations. First of all, the coating on the surface of the base material for a long time will cause different degrees of shedding, causing further infection. Second, surface modification increases the economic cost of biomaterials, and the modification process may pollute the environment. Lastly, higher complexity of the modification process also needs to be solved. In different strategies, there are differences in the interactions of human microenvironments. In fact, *in vivo* studies of surface-modified biomaterials are still lacking to further demonstrate their antibacterial properties and stability. The testing environment for the antimicrobial activity of a biological material should be relevant to the strains present in its application scenario. In order to guarantee the long-term stability of implants applied in orthopedics and dentistry under functional conditions, it is essential to test the mechanical properties of materials. In addition, the antibacterial properties, biocompatibility and other mechanisms of surface coatings or films in physiological environments need to be investigated. Surface-modified biomaterials have a good prospect for clinical applications, but there are still difficulties in the preparation and processing of materials in large-scale production and development, and the realization of clinical translation and large-scale production needs to be optimized in future research. Therefore, future recommendations in this field include reducing the additional cost of surface modification strategies, improving the stability of modified biomaterials, using facile approaches for further modifying biomaterials reasonably and achieving clinical translation.

## Conflicts of interest

The authors declare no conflict of interest.

## Supplementary Material
